# Profiling of 520 Candidate Genes in 50 Surgically Treated Chinese Small Cell Lung Cancer Patients

**DOI:** 10.3389/fonc.2021.644434

**Published:** 2021-06-08

**Authors:** Ting Yuan, Xin Wang, Sijin Sun, Zheng Cao, Xiaoli Feng, Yibo Gao

**Affiliations:** ^1^ Department of Pathology, National Cancer Center/National Clinical Research Center for Cancer/Cancer Hospital, Chinese Academy of Medical Sciences and Peking Union Medical College, Beijing, China; ^2^ Department of Thoracic Surgery, National Cancer Center/National Clinical Research Center for Cancer/Cancer Hospital, Chinese Academy of Medical Sciences and Peking Union Medical College, Beijing, China; ^3^ State Key Laboratory of Molecular Oncology, National Cancer Center/National Clinical Research Center for Cancer/Cancer Hospital, Chinese Academy of Medical Sciences and Peking Union Medical College, Beijing, China

**Keywords:** next generation sequencing, tumor mutation burden (TMB), small cell lung cancer (SCLC), signaling pathway, immunohistochemistry, immunohistochemistry

## Abstract

Small cell lung cancer (SCLC) is one of the severe malignancies with high mortality. Surgically resected tumor tissues from 50 Chinese SCLC patients were collected for next-generation sequencing to detect 520 cancer‐related genes. The most frequently altered genes were *TP53* (94.0%), *RB1* (86.0%), *LRP1B* (44.0%), *SPTA1* (26.0%) and *KMT2D* (24.0%). We detected that *NOTCH2, JAK2* and *CDK12* (*P*<0.05) had a significantly higher mutation frequency in Chinese SCLC compared to the Cologne and MSKCC. The single nucleotide variation (SNV) is dominated by C>A (34.1%). We found a significant association between TMB-H (≥10.3muts/Mb) and *ATM* (*P*=0.023), *CREBBP* (*P*=0.010), *KMT2D*(*P*=0.050) and *LRP1B* (*P*=0.005) gene mutations in Chinese SCLC patients. Immunostaining was performed using the following antibodies: TTF-1, CgA, CD56, Syn, and Ki-67. Correlation analysis between the expression of 6 markers and mutations in signaling pathways showed that Syn and CgA expression were associated with 4 (cGMP-PKG, Chemokine, TGF-β and Phospholipase D) and 2 (cGMP-PKG and Phosphatidylinositol) signaling pathway mutations. Kaplan-Meier curve showed that age<55 years, mutant *ARID2* and high TMB (≥7muts/Mb) were associated with a better prognosis, while the prognosis of patients with mutations in the Ras pathway was significantly improved. High TMB is an important prognostic factor for SCLC patients showed by multivariate analysis. In the combined cohort composed of current and two previous studies, survival analysis showed that SCLC patients with mutant *LRP1B* demonstrated better OS (*P*=0.0017). Patients with a high TMB (≥7muts/Mb) have a better prognosis (*P*=0.0053), consistent with our results in the Chinese cohort. We characterized the genomic alterations profile of Chinese SCLC patients and analyzed the correlation between genomic changes and immunohistochemical phenotypes at the signaling pathway level. Our data might provide useful information in the diagnosis and treatment for Chinese SCLC patients.

## Introduction

Lung cancer is the leading cause of cancer-related deaths worldwide. In China, there are approximately 787,000 newly diagnosed lung cancer and 631,000 deaths annually (1). Small cell lung cancer (SCLC) represents 13% of all newly diagnosed lung cancer cases worldwide (2). SCLC is characterized by a shorter tumor doubling time, and a higher rate of early distant metastasis compared to non-small cell lung cancer (NSCLC). According to the staging criteria of the American Veterans Lung Cancer Association (VALG), SCLC patients can be divided into limited disease (LD) and extensive disease (ED). Chemotherapy and radiotherapy are the main treatment options for SCLC, doctors often use comprehensive treatment according to the patient’s condition and the tumor stage. SCLC patients often show an initial response to the treatment, but most of them inevitably develop recurrence and metastasis due to drug resistance. Therefore, it is crucial to actively explore the second-line and even third-line treatment approaches.

Immunotherapy has revolutionized the treatment of SCLC. Atezolizumab is a humanized monoclonal anti–programmed death ligand 1 (PD-L1) antibody that inhibits PD-L1–programmed death 1 (PD-1) and PD-L1–B7-1 signaling and restores tumor-specific T-cell immunity (3). U.S. Food and Drug Administration (FDA) approved Atezolizumab in combination with carboplatin and etoposide as first-line treatment for patients with ED-SCLC. This is the first immune checkpoint inhibitor (ICI) approved as a first-line treatment for SCLC (4, 5). With the advancements in sequencing technologies and our understanding of SCLC, it is necessary to comprehensively profile SCLC to reveal potential druggable targets. It has been reported that a few cases of never-smokers SCLC patients harboring epidermal growth factor receptor (*EGFR*) mutations, and responded to EGFR tyrosine kinase inhibitors (TKIs) (6). Bi‐allelic inactivation of *TP53* and *RB1* can be detected in nearly all SCLC tumors, suggesting that loss of the tumor suppressors *TP53* and *RB1* is obligatory in SCLC. Other mutations, including *TTN, RYR2, LRP1B, MUC16, ZFHX4, USH2A, CSMD3, NAV3, PCDH15* and *COL11A1* were also identified in SCLC (7). Based on next-generation sequencing (NGS) technology, comprehensive genomic profiling of tumors can detect genetic changes from point mutations to large structural variations.

In this study, we profiled 50 Chinese SCLC patients’ surgically resected tumor sample using a panel of 520 cancer-related genes. Sequencing results, immunohistochemical expression and clinical data were analyzed. We identified some unique genetic variant features in Chinese SCLC patients and found that somatic co‐occurring *TP53/RB1* mutations were frequent in Chinese SCLC patients. We further identified the correlation between immunohistochemistry (IHC) markers and gene mutations in several cancer-related signaling pathways. Univariate and multivariate analyses were performed to determine patient survival. These results might be helpful in the diagnosis and treatment of Chinese SCLC patients.

## Materials and Methods

### Patients and Sample Collection

This single-center retrospective study included 50 SCLC patients who were diagnosed and received surgery at the Cancer Hospital of the Chinese Academy of Medical Sciences from 2010 to 2012. After obtaining informed consent, tissue samples were retrieved from the Department of Pathology. The Institutional Review Board (IRB) approved the collection and use of all specimens in this study. In this study, the clinical characteristics of patients were also retrieved. Capture-based targeted sequencing was performed by Burning Rock Biotech, Guangzhou. DNA of FFPE samples was extracted using a QIAamp DNA FFPE tissue kit (QIAGEN, Valencia, CA). DNA concentration was measured by Qubit dsDNA assay. The gDNA quality was assessed to ensure that A260/A280 is within the range of 1.8 to 2.0.

### Library Preparation and Sequencing

A commercially available panel consisting of 520 cancer-related genes (**Table S1**), spanning 1.67M of the Human genome was used for molecular profiling. An input DNA of 40 ng was required. Fragments of 200 to 400-bp sizes were selected with beads (Agencourt AMPure XP kit; Beckman-Coulter, Brea, CA), followed by hybridization with the capture probes baits, hybrid selection with magnetic beads, and PCR amplification. Then, a bioanalyzer high-sensitivity DNA assay was used to assess the quality and size range. Next, we sequenced available indexed samples on a Miseq (Illumina, San Diego, CA) with pair-end reads. We sequenced only tumor DNA without normal match DNA for each patient. Therefore, we selected putative somatic mutations using the following filters: (1) The maximum population frequency (1000 Genomes, ExAC, gnomAD exome, gnomAD genome) ≥ 0.001 is germline variation; (2) The pathogenicity rating is B/LB/VUS and population frequency is greater than 0 and AF frequency is between 35% and 65%.

Sequencing data were mapped to the human genome (hg19) using BWA aligner 0.7.10. PCR duplicate reads were removed before base substitution detection. Local alignment optimization and variant calling was performed using GATK v3.2-2. DNA translocation analysis was performed using Tophat2 and Factera 1.4.3. Insert size distribution and library complexity of each sample were computed to assess the level of DNA degradation. Different mutation calling thresholds were applied on samples with different DNA quality to avoid false-positive mutation calls due to DNA damage. SNV and indels identified were annotated using the dbNSFP(v30a), COSMIC (v69), and dbSNP (snp138) database. Variants with a global minor allele frequency greater than 1.0% in the 1000Genome Project (Phase3, http://www.1000genomes.org/data) were considered common SNPs and removed. Integrative Genomics Viewer (Broad Institute, USA) was used to visualize variants aligned against the reference genome to confirm the accuracy of the variant calls by checking for possible strand biases and sequencing errors. Gene-level copy number variation (CNV) was assessed using a statistic after normalizing read depth at each region by total read number and region size and correcting GC-bias using a LOESS algorithm.

The reported mutations were further confirmed using dbSNP, ClinVar databases and Intervar (8), the computational tool for semi-automated variant interpretation, which was used to aggregate the variant annotations from multiple databases, prediction tools and publications at a single site. In the absence of clinical data and *in vitro* functional assay, in silico predictions using algorithms that assess phylogenetic conservation and the likelihood of severe physiochemical alterations in the protein structure or function were utilized as prediction tools. All genetic annotations and nomenclature were based on GRCh37/hg19 build. According to the American Society of Medical Genetics and Genomics (ACMG), the variants were classified according to standards of interpretation and reporting of sequence variations. The variants were organized into five classes as follows: pathogenic (Class 5), likely pathogenic (Class 4), variants of uncertain significance (Class 3), likely benign (Class 2) and benign (Class 1) (9). Without departing from the scope of this study, we only analyzed pathogenic and likely pathogenic (LP/P) mutations.

### Immunohistochemical (IHC) Staining

Immunostaining was performed on an automatic immunostaining machine using the following antibodies: TTF-1, chromogranin A, CD56, CK18, synaptophysin and Ki-67 according to the manufacturer’s instructions. The sources and positive sites of primary antibodies were shown in **Table S2**. Negative and positive controls were included in each batch. For each antibody, the percentage of positive cells and the intensity of staining (0: negative; 1+: weak; 2+: moderate; 3+: strong) were recorded. For Ki-67, the percentage of positive tumor cells was recorded as 0-25%, 25-50%, 50-75%, and 75-100%. A tumor was considered positive for a specific marker when at least 10% of the neoplastic cells reacted with an intensity of 2+ or greater on the relevant subcellular localization.

### Follow-Up and Statistical Analysis

Patients were followed up prospectively *via* routine hospital visits or telephone calls. The phone calls were conducted by trained medical staff with the patients or their family members once every three months until death or last follow-up. The median follow-up time for this cohort was 44.8 months (range 1.6-106.5 months), and 16% of the patients were alive at the time of the last follow-up. Overall survival (OS) was measured from the day of SCLC diagnosis to the day of death of any cause and was analyzed by Kaplan– Meier estimates and log-rank test. For variables with a *P*-value of 0.1 or less in univariate analysis, a multivariate analysis was carried out using Cox’s regression analysis. Correlations between categorical variables were calculated using χ2 and Fisher’s exact tests. Statistical analyses were performed using SPSS 23.0, and *P* ≤ 0.05 was considered to be statistically significant.

## Results

### Patient’s Characteristics

The main clinical features of the cohort are summarized in **Table 1**. The median age of the cohort was 57 years (range 28-78) with a majority of them being male (72.0%). Thirty-two patients (64.0%) were smokers. Furthermore, 30 patients (60%) had central type; 8 patients (16%) were stage I, 15 (30%) were stage II and 24 (48%) were stage III. These 50 cases are all surgically resected samples. All the genes within the detection range are included in the TMB (tumor mutation burden) calculation, excluding the kinase region mutations of *EGFR* and *ALK*, and the total number of CNV and fusion. TMB is defined as mutations of the corresponding genome interval size. Median non-synonymous TMB was 10.3 muts/Mb (range 4.0-23.0 muts/Mb). Eight patients (16%) received CE (carboplatin and VP-16) chemotherapy and 26 patients (52%) received EP (cisplatin and VP-16) chemotherapy. 12 patients (24%) had radiotherapy, of whom 1 with prophylactic cranial irradiation (PCI).

**Table 1:** Patients’ characteristics.

Characteristics	Frequency (n=50)	Percentage
Age		
Median [range], years	57 [28-78]	
Sex		
Male	36	72.0
Female	14	28.0
Smoking status		
Smokers	32	64.0
Never-smoker	17	34.0
Unknown	1	2.0
Type		
Central	30	60.0
Peripheral	20	40.0
Stage		
I	8	16.0
II	15	30.0
III	24	48.0
Unknown	3	6.0
TMB		
Median [range], muts/Mb	10.3[4.0-23.0]	
<10.3 muts/Mb	24	48.0
≥10.3 muts/Mb	26	52.0
Chemotherapy		
CE	8	16.0
EP	26	52.0
Unknown	16	32.0
Radiotherapy		
Local	11	22.0
PCI	1	2.0
No	17	34.0
Unknown	21	42.0

### Comprehensive Genomic Profiles of SCLC

**Figure 1:**
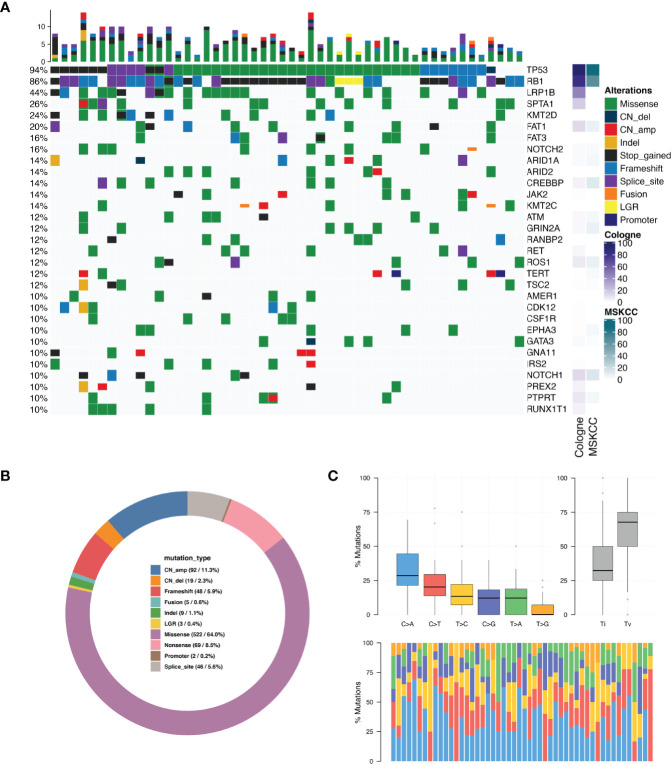
Mutation spectrum in SCLC tumors. **(A)** Genomic profiling. The abscissa was the tumor specimens and the mutations detected for a given gene is shown on the right axis. Dark green bars indicate missense mutations, dark blue indicate copy number deletion, light red indicate copy number amplification, dark orange indicate small insertions or deletions, black indicate stop gained mutations, light blue indicate frame-shift mutations, purple indicate splice site mutations, bright orange indicate gene fusions, yellow indicate large genomic rearrangement, indigo blue indicate promoter region variant. The percentage of total patients with any type of mutation in a given gene is shown on the left axis. The results are shown only for genes which were mutated in N > 4 patients. **(B)** Distribution of mutations in SCLC tumors. **(C)** Single nucleotide variation analysis.

Collectively, we identified 961mutations and the most frequently mutated gene were *TP53* (N = 47, 94.0%), *RB1* (N = 43, 86.0%), *LRP1B* (N = 22, 44.0%), *SPTA1* (N = 13, 26.0%) and *KMT2D* (N = 12, 24.0%) (**Figure 1A**). **Figure 1A** shows genes that were found to be altered in at least five patients (see **Table S3** for mutated amino acid substitution information). *MYCN* missense mutation was detected in two patients (4%) and *SOX2* copy number amplification was detected in one patient (2%). We compared the mutation frequency of SCLC between the current Chinese cohort and Cologne and MSKCC cohorts (7, 10), whose patients come from Europe and North America, respectively. As envisioned,the mutation frequency of *TP53* (94%) was similar to those two studies of Caucasian ethnics (85.8% and 86.6%). However, the mutation rate of *RB1* in our Chinese cohort was similar to the Cologne cohort (86% vs 79.1%, P=0.299, Fisher’s exact test) but higher than the MSKCC cohort (86% vs 65%, P=0.007, Fisher’s exact test). *NOTCH2* mutations occurred in 16% of Chinese SCLC patients, whereas they displayed a prevalence of 4.5% (*P*=0.025) in the Cologne database and 3.7% (*P*=0.02) in the MSKCC database. In addition, we found that the mutation frequencies of *JAK2* (14% vs. 2.7% and 1.2%, *P*<0.01) and *CDK12* (10% vs. 1.8% and 1.2%, *P*<0.05) in the Chinese cohort were significantly higher than in Cologne and MSKCC. Collectively we detected 3 cancer genes that have a significantly higher mutation frequency in Chinese SCLC compared to the other two cohorts. The percentage of the different types of mutations detected among the 50 patients is shown in **Figure 1B**. The single nucleotide variation (SNV) was dominated by C>A, accounting for 34.1% of the total SNVs. Others, C>G was 11.8%, C>T was 23.3%, T>A was 11.7%, T>C was 14.8%, and the lowest frequency of SNV T>G was 4.4% (**Figure 1C**). The details of CNV are listed in **Table S4**. C>A transversions occurred most frequently, consistent with the association of tobacco smoking with the occurrence of C>A transversions (11).

### Tumor Mutation Burden Analysis

**Figure 2:**
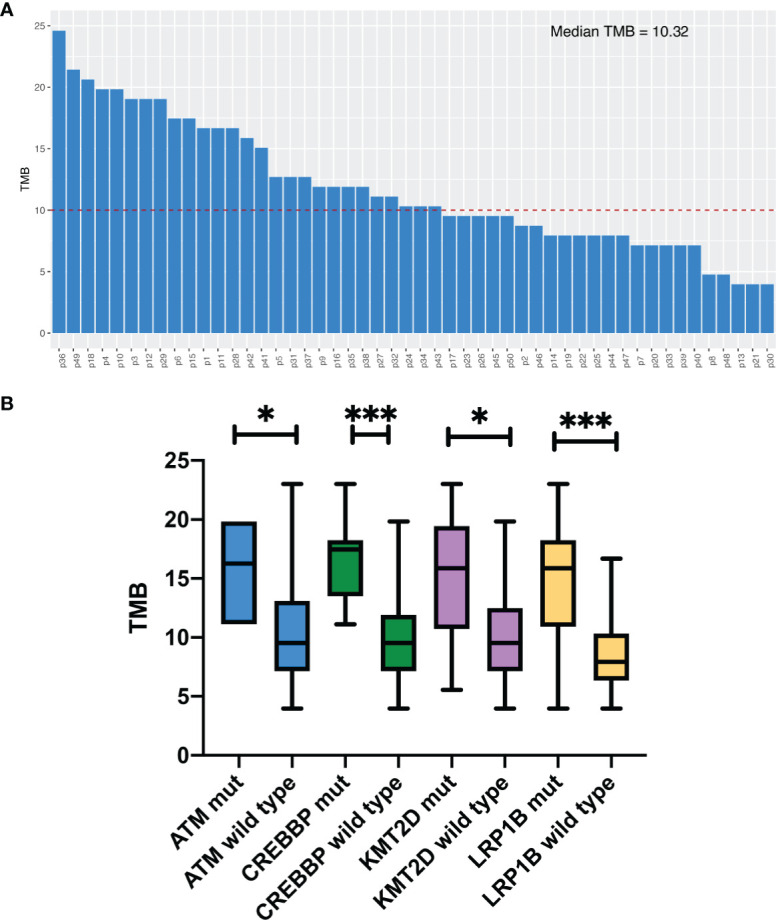
Tumor mutation burden analysis. **(A)** TMB levels in 50 patients. **(B)** Comparison of median. TMB in Chinese SCLC patients with certain specific gene mutations. *P<0.05, ***P<0.001.

Median TMB for the entire study population was 10.3 muts/Mb (range: 4.0–23.0). The details of the TMB are shown in **Figure 2A**. We divided the patients into two groups according to the TMB level, TMB-H (≥10.3muts/Mb) and TMB-L (<10.3muts/Mb). By Fisher’s exact test, we found a significant association between TMB‐H and gene mutations, including *ATM, CREBBP, KMT2D* and *LRP1B*, in the Chinese SCLC patients (**Table S5**). The mutation rates of these genes mentioned above in the TMB‐H group were much higher than the TMB‐L group. In addition, the median TMB of patients with these mutations was higher than those without the mutations (**Figure 2B**).

### Correlation Analysis of Immunohistochemistry and Gene Mutations in Cancer-Related Signaling Pathways

**Figure 3:**
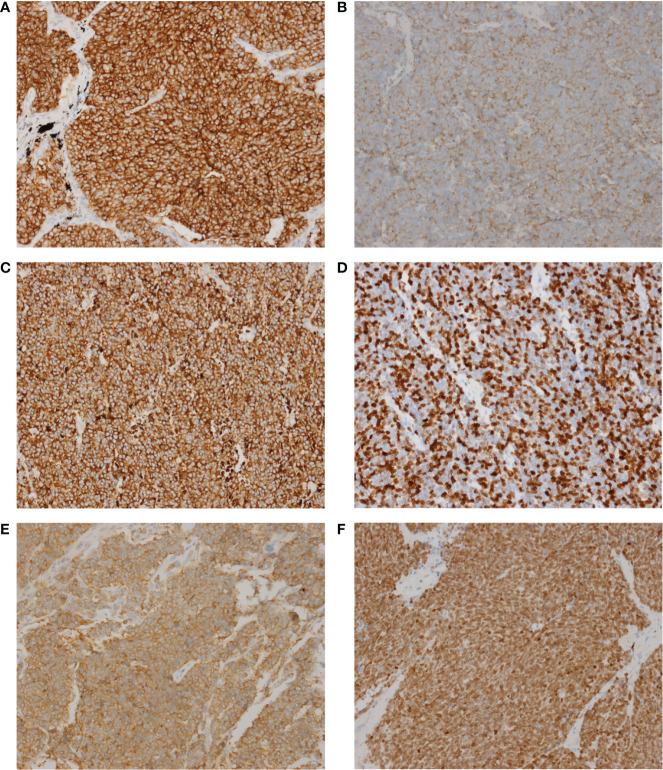
The representative images of these 6 immunohistochemical markers. **(A)** CD56. **(B)** CgA. **(C)** CK18. **(D)** Ki-67. **(E)** Syn. **(F)**, TTF-1. Magnification, x200.

Since SCLC is classified with neuroendocrine tumors, immunohistochemistry is very important in the diagnosis of SCLC, especially some neuroendocrine markers, such as Synaptophysin (Syn), Chromogranin A (CgA) and CD56 (an alias for neural cell adhesion molecule 1) (12). Here, we performed 6 immunohistochemical staining on these 50 SCLC patients, including Syn, CgA, CD56, TTF-1, CK18 and Ki-67. These six markers are routinely reported by the pathology department of our hospital and other academic clinical centers, which are recommended by NCCN Guidelines (Small Cell Lung Cancer, Version 1.2021 -August 11, 2020). However, the relationship between these markers and cancer gene mutations had never been reported. The relationship between them and the signaling pathway has not been thoroughly explored. The representative images of these 6 immunohistochemical markers are shown in **Figure 3**. The expression of the above 6 markers is shown in **Table 2**.

**Table 2:** IHC and molecular analysis.

Markers	N	Percentage (%)
Syn		
Negative	2	4.0
1+	13	26.0
2+	26	52.0
3+	9	18.0
CgA		
Negative	6	12.0
1+	19	38.0
2+	21	42.0
3+	4	8.0
CD56		
Negative	1	2.0
1+	3	6.0
2+	7	14.0
3+	39	78.0
TTF-1		
Negative	5	10.0
1+	5	10.0
2+	8	16.0
3+	32	64.0
CK18		
Negative	2	4.0
1+	17	34.0
2+	19	38.0
3+	12	24.0
Ki-67		
0-25%	9	18.0
25-50%	6	12.0
50-75%	26	52.0
75-100%	9	18.0

The KEGG (http://www.genome.ad.jp/kegg/) database collects genomic, chemical, and systematic functional information (13). PathScan (14) analysis was performed to identify significant clusters of point mutations involving genes in annotated KEGG (Kyoto Encyclopedia of Genes and Genomes) pathways. The correlation between the expression of 6 immunohistochemical markers and pathway mutations shows that Syn expression is related to mutations in 4 signaling pathways (cGMP-PKG, Chemokine, TGF-β and Phospholipase D), while CgA is related to 2 signaling pathway (cGMP-PKG and Phosphatidylinositol). The number of genetic mutations in each pathway is shown in **Table 3**.

**Table 3:** Correlation analysis of immunohistochemistry (IHC) and gene mutations in cancer-associated signaling pathways.

Markers	Pathway	Mutation genes (of mutated cases)	R	*P* Value
Syn	cGMP-PKG	AKT1(1), GNA11(5), GNAQ (1), INSR (2), IRS1(1), IRS2(5), KRAS (2), MAP2K1(1), MAP2K2 (4), MAPK3(1), MEF2B (2), PIK3CG (4)	0.400	0.005
	Chemokine	AKT1(1), CXCR4(1), GSK3B (1), JAK2(7), KRAS (2), MAP2K1(1), MAPK3(1), NFKBIA (1), PIK3CB (1), PIK3CG (4), SRC (1)	-0.297	0.036
	TGF-β	BMPR1A (1), CDKN2B (1), CREBBP (7), EP300 (4), GREM1(1), ID3 (1), INHBA (1), MAPK3(1), RPS6KB2(1), SMAD2(2), SMAD4(2), TGFBR2(2)	-0.297	0.036
	Phospholipase D	INSR(2), KIT(1), KRAS(2), MAP2K1(1), MAP2K2(4), MAPK3(1), MTOR(4), PDFDRA(3), PDGFRB(2), PIK3CB(1), PIK3CG(4), PIK3R1(1), PLCG2(4), PTPN11(1)	0.280	0.048
CgA	Chemokine	AKT1(1), CXCR4(1), GSK3B (1), JAK2(7), KRAS (2), MAP2K1(1), MAPK3(1), NFKBIA (1), PIK3CB (1), PIK3CG (4), SRC (1)	-0.371	0.009
	Phosphatidylinositol	PIK3CB (1), PIK3R (1), PTEN (3)	-0.333	0.050

### Survival Analysis

**Figure 4:**
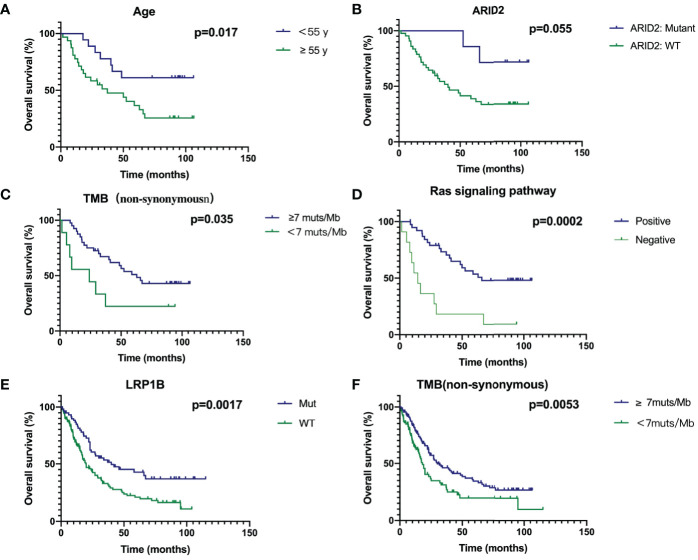
Survival analyses of Age **(A)**, ARID2 **(B)**, TMB **(C)**, Ras signaling pathway **(D)**, LRP1B **(E)** and TMB in combined cohort **(F)** through Kaplan‐Meier curve with log‐rank test.

The Kaplan-Meier estimate curves of patients stratified by the age of diagnosis of SCLC are shown in **Figure 4A**. The OS of patients<55 years old was statistically longer than that of patients≥55 (Hazard Ratio 0.3736, 95% CI 0.1800–0.7755, *P* = 0.017). *ARID2* mutation status had the most significant impact in any gene, as SCLC patients with wild-type *ARID2* demonstrated worse OS than the patients with mutant *ARID2* (HR 3.664, 95% CI 1.507–8.906, *P*=0.055) (**Figure 4B**). No other genes were prognostic in patients we studied. Furthermore, SCLC is a tumor with high TMB, which may be due to a history of smoking in most patients. Our results indicate that patients with a high mutation TMB (≥7muts/Mb) have a better prognosis (HR 0.4131, 95% CI 0.1326-1.286, *P*=0.035) (**Figure 4C**).

If a gene contained in a signal pathway is mutated, we define this signal pathway as positive, otherwise it is defined as negative. Genes in the Ras pathway was defined as genes identified and annotated in KEGG (http://www.genome.ad.jp/kegg/) database, which did not refer to *KRAS* hotspot mutations. In **Supplementary Figure 1**, genes with mutations in the RAS signaling pathway are shown in red, and green is the background color. Patients with mutations in Ras signaling pathway had a significantly better prognosis (HR 0.2567, 95% CI 0.08343-0.7899, *P* = 0.0002) (**Figure 4D**). Due to the small number of patients in our cohort, we combined the previous two studies and our cohort to analyze prognostic factors (7, 10). Thus, the total number of patients had reached 252. In our cohort of 50 patients, survival curves for *LRP1B* were not statistically significant (*P*=0.1751, **Supplementary Figure 2**). However, survival analysis in the combined cohort showed that SCLC patients with mutant *LRP1B* demonstrated better OS (*P*=0.0017) (**Figure 4E**). The cut-off points of TMB (7muts/Mb) in our cohort also applied to the combined cohort. Patients with a high mutation TMB (≥7muts/Mb) have a better prognosis (*P*=0.0053) (**Figure 4F**), which is consistent with our results in the Chinese cohort.

Using multivariate analysis to detect an independent effect, we identified the classic known prognostic factors in SCLC patients in our cohort, such as patients with advanced stage having a worse prognosis (HR 3.005, 95% CI 0.98-9.216, *P* = 0.054). *P >*0.05 may be due to the small number of patients in our cohort. In addition, high TMB was a significant prognostic factor in SCLC patients (*P* = 0.034) with a hazard ratio (comparing low TMB) of 0.251 (CI 0.070-0.901) (**Table 4**).

**Table 4:** Variables accepted in the backward selection model of the Cox regression as explanatory factors.

Variables	HR	95% CI	*p value*
Age	3.474	1.292-9.341	0.014
Stage	3.005	0.98-9.216	0.054
TMB (non-synonymous)	0.251	0.070-0.901	0.034

## Discussion

SCLC accounts for about 13% of the total number of lung cancers. Its main characteristics are the early invasion and metastasis and high sensitivity to primary chemotherapy. Although it responds better to the initial treatment, almost all patients inevitably develop treatment resistance, causing disease progression. Therefore, SCLC is still a highly lethal malignant tumor. The recent rise of targeted therapies has brought a new gospel to cancer patients, but the genetic analysis of SCLC is challenging. The SCLC patients have little chance of receiving surgical treatment, which largely determines the lack of sufficient tissue samples for comprehensive genetic analysis. The history of research and development of EGFR-TKI and ALK inhibitors in lung cancer treatment reveals that finding clear driver genes for tumors is of great significance for developing new drugs, selecting the right treatment population and ensuring the therapeutic efficacy of targeted drugs. Therefore, it is imperative to comprehensively analyze the genomics characteristics of SCLC using NGS.

In this study, we collected 50 surgical samples of SCLC patients and examined the genomic alterations. Most of the existing reports of SCLC are advanced patients, and there are few studies on limited patients like ours. The profile was produced using a capture-based targeted sequencing panel that target 520 genes and spanning 1.67M of Human genomic regions. In addition to *TP53* and *RB1*, the genes with high mutation frequencies also detected in the previous SCLC studies, including *LRP1B* (44.00%), *SPTA1* (26.00%), *FAT1*(20%), *ARID1A* (14%), *CREBBP* (14.00%), *ROS1* (12.00%), and *PTPRT* (10.00%) (7, 10). *MYC* is altered in 5.49% of SCLC patients with *MYC* amplification present in 4.58% of all SCLC patients (15). However, in our cohort *MYCN* missense mutation was detected in only 2 patients, and no *MYC* amplification was detected. Considering the relatively low proportion of *MYC* amplification in SCLC patients, it is reasonable to not be detected in our cohort of 50 patients. *SOX2* copy number amplification was detected in one patient (2%), which are consistent with the mutation frequency of previous studies (*SOX2* mutated 5% in Multi-Institute, Cancer Cell 2017) (16). We compared the mutation frequency of SCLC between our cohort and Cologne and MSKCC cohorts. As envisioned, the mutation frequency of TP53 (94%) was similar to those two studies of Caucasian ethnics (85.8% and 86.6%). However, the mutation rate of RB1 in our Chinese cohort was similar to the Cologne cohort (86% vs 79.1%, P=0.299, Fisher’s exact test) but higher than the MSKCC cohort (86% vs 65%, P=0.007, Fisher’s exact test). Collectively we detected 3 cancer genes that have a significantly higher mutation frequency in Chinese SCLC than the other two cohorts. It is worth noticing that both the Cologne cohort and MSKCC cohort are from Caucasian ethnics but undergone different sequencing techniques. Thus, the variation in mutation frequency across cohorts may be due to differences in technical details or bioinformatic analysis. For TMB, the figures for Cologne and MSKCC are 7.98 muts/Mb and 4.99 muts/Mb respectively, while the TMB in our study is high as 10.32 muts/Mb. The 520 panel simultaneously detects targeting, drug resistance mechanism, MSI, TMB and other immune biomarkers. The average sequencing depth of our panel is 1000X, higher than that of the MSK Panel, which may lead to better sensitivity. Consistent with these two studies, the most frequent mutation and SNV type is missense mutation and C>A. According to the results of previous research by Johannes Voortman et al. (17), we detected a high copy number of *JAK2* (4%) in samples of SCLC, suggesting that it may represent a driving mutation gene for some SCLC, so that it may become a target for treatment.

SCLC tumors had a significantly high mutation rate (TMB‐H), which is considered as a biomarker of response to immunotherapy in SCLC, and immunotherapy may improve SCLC treatment (18). However, the response rate of patients with SCLC to immunotherapy is much lower than the proportion of patients with TMB‐H, suggesting that more biomarkers may be required in SCLC. In this study, we found that the mutation rates of *ATM, CREBBP, KMT2D* and *LRP1B* in the TMB‐H group were significantly higher than those in the TMB‐L group. The Cologne and MSKCC studies supported the findings of *LRP1B* (*P ≤* 0.001) and *CREBBP* (*P*<0.038), respectively. Mutations in *CREBBP* and *EP300* were significantly clustered around the sequence encoding the histone acetyltransferase (HAT) domain (19). In addition, mutations in *CREBBP, EP300, TP73, RBL1, RBL2* and *NOTCH* family genes are largely mutually exclusive, suggesting that they may play similar tumor-promoting roles in the onset of SCLC (7). Survival analysis in the combined cohort showed that SCLC patients with mutant LRP1B demonstrated better OS. In NSCLC, over-expression of full-length LRP1B results in impaired cell proliferation, consistent with hypothetical tumor suppressor function, while LRP1B knockout is the opposite (20). Considering the role of *LRP1B* and *CREBBP* in tumors, the significance is much less clear and functional experiments will be required, but it may provide more possibilities on developing new factors that predict response to immune checkpoint inhibitor therapy.

We performed immunohistochemical staining on these 50 patients with SCLC, including Syn, CgA, CD56, TTF-1, CK18, and Ki-67. Correlation analysis between the expression of 6 immunohistochemical markers and mutations in signaling pathways showed that Syn and CgA expression were respectively associated with 4 (cGMP-PKG, Chemokine, TGF-β and Phospholipase D) and 2 (cGMP-PKG And Phosphatidylinositol) signaling pathway mutations. This suggests that the immunophenotypic characteristics of SCLC may be related to the gene mutations enriched into the signaling pathway so that it can be studied and verified in the future by experiments.

Kaplan-Meier curve showed that age<55 years, mutant *ARID2* and TMB-H were associated with a better prognosis, while the prognosis of patients with mutations in the Ras pathway was significantly improved. We found that the gene mutation in the Ras pathway was associated with a better prognosis, indicating that instead of the hotspot mutations with constitutional activation of kras protein, other genes in this important pathway, especially those downstream of Ras, may have other effects or mechanisms. The prognostic features of AIRD2 and RAS signaling pathways are only identified in the Chinese cohort, reflecting the relevance for Chinese patients. The study of Ohashi et al. (21) has shown that mutations in RAS pathway signaling genes may be infrequent but occur and could influence therapeutic outcomes in trials for lung cancer patients with acquired resistance to EGFR TKIs. Khanzada, U. K. et al. (22) demonstrated that inhibiting the Ras signaling pathway with simvastatin potently disrupts growth and survival in human SCLC cells. These two studies are consistent with our results. Therefore, specific studies on the *ARID2* gene and Ras pathway can be conducted in the future. Using multivariate analysis to detect independent effects, we identified the classic known prognostic factors for SCLC patients in the cohort, such as poor prognosis in high-stage patients. In addition, high TMB is an important prognostic factor for SCLC patients.

There is some limitation to the current study. For example, the number of cases is not enough, and the detection of the gene Panel cannot fully reflect the gene mutation, which may impact the result. In summary, we found genomic characteristics and association with immunophenotypes in Chinese patients with SCLC. Our data may provide useful information for the diagnosis and treatment of SCLC patients in China.

## Data Availability Statement

All datasets generated for this study are included in the article/**Supplementary Material**.

## Ethics Statement

The studies involving human participants were reviewed and approved by the Cancer Hospital of the Chinese Academy of Medical Sciences Institutional Review Board. The patients/participants provided their written informed consent to participate in this study.

## Author Contributions

XF and YG provided a holistic approach and put forward suggestions for revision of the paper. TY designed and performed research, analyzed and interpreted data, and wrote the manuscript. SS and XW analyzed and interpreted data. ZC selected and reviewed the cases. XF and YG contributed equally to this work. All authors contributed to the article and approved the submitted version.

## Funding

This study was supported by the National Key R&D Program of China (2018YFC1312100), Beijing Municipal Science & Technology Commission (Z191100006619117), CAMS Initiative for Innovative Medicine (2017–I2M–1–005, 2019–I2M–2–002), Nonprofit Central Research Institute Fund of Chinese Academy of Medical Sciences (2018PT32033), Innovation team development project of Ministry of Education (IRT_17R10).

## Conflict of Interest

The authors declare that the research was conducted in the absence of any commercial or financial relationships that could be construed as a potential conflict of interest.
