# Supplementary material for: Profiling of 520 Candidate Genes in 50 Surgically Treated Chinese Small Cell Lung Cancer Patients
Source: Front Oncol. 2021 Jun 8;11:644434. doi: 10.3389/fonc.2021.644434 (PMC8217828; doi:10.3389/fonc.2021.644434)
Supplement: Supplementary file 1 [file DataSheet_1.docx]

Supplementary Figure 1. Ras signaling pathway marked with mutant genes.


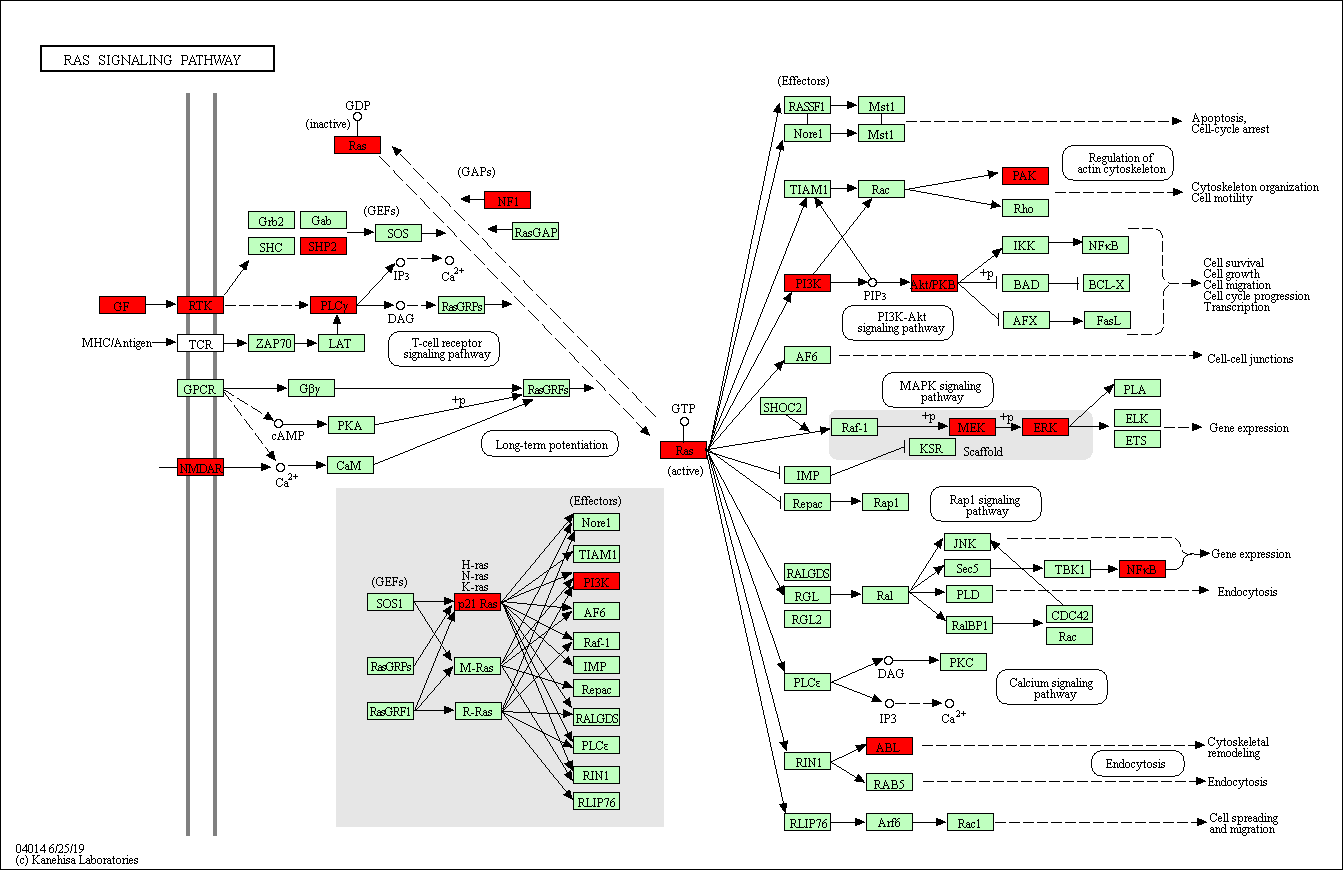
 Supplementary Figure 2. The survival data comparing LRP1B WT vs. mutant in the original cohort (50 cases).
